# Prevalence Study of MASLD in Adolescent and Young Adult Pacific Islanders and Asians Living in Hawai’i

**DOI:** 10.1210/jendso/bvad165

**Published:** 2024-01-18

**Authors:** Alan A Parsa, Katie A Azama, May Vawer, Mel A Ona, Todd B Seto

**Affiliations:** John A. Burns School of Medicine, University of Hawai’i, Honolulu, HI 96813, USA; Diabetes Research and Education Center of the Pacific, Honolulu, HI 96813, USA; Department of Medicine, The Queen's Medical Center, Honolulu, HI 96813, USA; Department of Medicine, The Queen's Medical Center, Honolulu, HI 96813, USA; Nancy Atmospera-Walch School of Nursing, University of Hawai’i, Honolulu, HI 96822, USA; Diabetes Research and Education Center of the Pacific, Honolulu, HI 96813, USA; Department of Medicine, The Queen's Medical Center, Honolulu, HI 96813, USA; John A. Burns School of Medicine, University of Hawai’i, Honolulu, HI 96813, USA; John A. Burns School of Medicine, University of Hawai’i, Honolulu, HI 96813, USA; Department of Medicine, The Queen's Medical Center, Honolulu, HI 96813, USA

**Keywords:** Hawai’i, NAFLD, MASLD, Native Hawaiian, Pacific Islander, Asian, nonalcoholic fatty liver, Hawaii

## Abstract

**Context:**

Nonalcoholic fatty liver disease, renamed metabolic dysfunction-associated steatotic liver disease (MASLD), is the most common cause of chronic liver disease with an estimated worldwide prevalence of 30.1% while clinical practice observations reflect a disproportionately lower prevalence of 1.9%, indicating a condition that is underrecognized in clinical care settings. Screening for MASLD is rarely performed, and little is known about the prevalence in Hawai’i.

**Objective:**

This pilot aims to develop an understanding of the prevalence and factors associated with MASLD in Hawai’i's adolescent and young adult (AYA) population.

**Design/Methods:**

Cross-sectional observational pilot study: We used Fibroscan^®^—liver ultrasonographic vibration-controlled transient elastography (VCTE) to identify MASLD based on controlled attenuation parameter (CAP) scores ≥238 (dB/m) and collected biometric, anthropometric, and Beverage Intake Questionnaire (sugar-sweetened beverage) survey data.

**Setting:**

The study took place at community clinics in Hawai’i on the island of O’ahu.

**Participants:**

One hundred individuals were evaluated, age 14 to 34 years.

**Main Outcome Measures:**

We used VCTE Fibroscan^®^ with CAP scoring to identify the presence of hepatocyte steatosis (fatty liver).

**Results:**

Overall MASLD prevalence in the sample was 44% (95% confidence interval: 34.1%-54.3%). In participants with MASLD, obese Native Hawaiian and other Pacific Islanders (62%) and nonobese Asians (43%) had the highest rates of MASLD.

**Conclusion:**

This pilot evaluation of the AYA NHOPI and Asian MASLD population in Hawai’i shows a higher rate of MASLD than those reported in other parts of the United States. Larger population health studies are indicated to expand our knowledge of MASLD in the Hawaiian Islands.

Nonalcoholic fatty liver disease, recently renamed metabolic dysfunction-associated steatotic liver disease (MASLD) [1], is the most common liver condition in the world and is divided into 2 forms: nonalcoholic fatty liver, renamed steatotic liver disease, which describes fatty liver without inflammation and nonalcoholic steatohepatitis, renamed as metabolic dysfunction-associated steatohepatitis (MASH), the more severe phenotype with hepatocellular inflammation. Both steatotic liver disease and MASH can progress to fibrosis with nonalcoholic steatohepatitis being the more aggressive form [2] in which 9% to 25% develop cirrhosis over 10 to 20 years [3]. The resultant is liver failure or hepatocellular carcinoma (HCC), which may lead to liver transplantation [3, 4]; in fact, MASLD was the second most common indication for liver transplant in 2019 [4]. The global prevalence of MASLD has been rapidly increasing from 25% in 2018 [5] to 30% in 2023 [6]. In the United States, the reported prevalence of MASLD in adults is 32.3% [7]. A 2021 study by Yu and Schwimmer suggests a likely global prevalence of MASLD in children to be between 5% and 10% [8] with other studies reporting a range between 2.5% and 45% depending on ethnicity, age, and obesity status [9, 10]. In the US youth population, MASLD prevalence ranges between 13.0% and 24.0% [9, 10]. An estimated 10% to 20% of children with MASLD will progress to advanced fibrosis [8].

Currently, a global knowledge gap exists in both primary care physicians and endocrinologists for the identification, diagnosis, and management of MASLD [11]. Society recommendations reserve MASLD screening for individuals with obesity and type 2 diabetes mellitus (T2DM) [12, 13]. These are high risk conditions strongly correlating with other (ie, cardiac, vascular, and renal) complications that practitioners may take precedence in treating. Global chart review in the nonacademic setting shows a MASLD prevalence of 1.9% [14], compared to the published 30% worldwide prevalence [6], consistent with low published rates of screening for MASLD [15]. This indicates a condition that is significantly underrecognized in clinical care settings. In Hawai’i, MASLD prevalence data is practically nonexistent. A high prevalence of obesity in Native Hawaiians (43.7%) and other Pacific Islanders (59.4%) [16], along with a high incidence of T2DM among Native Hawaiians and Other Pacific Islander (NHOPI) children (7.7% per year) [17], places them at high risk for advanced liver disease. In the NHOPI and Asian populations who encompass two-thirds of the state's population [18], the prevalence of MASLD remains relatively unknown, especially in the young population without diabetes. Identifying MASLD in adolescents and young adults (AYA) is critical for early intervention and prevention of future complications including need for transplantation. To gain a foundational understanding of the prevalence and factors associated with MASLD in Hawai’i's AYA population, we conducted a prevalence pilot study evaluating 100 participants, predominantly NHOPIs and Asians living in Hawai’i on the island of O’ahu.

## Methods

### Study Population

Participants were all residents of the island of O’ahu and recruited through local advertising at school campuses and word-of-mouth (95% of participants) and primary and specialty care clinics (5% of participants). The majority of participants were recruited from the University of Hawai’i and by word-of-mouth among students. Eligible individuals included males and females between 14 and 34 years old, who were able to provide written informed consent (or assent if applicable). Adolescent was defined as those aged 14 to 19 years (n = 16) and young adults were 20 to-34 years (n = 84). Obese was defined by a body mass index (BMI) of ≥30 while nonobese was defined as a BMI < 30. We excluded those with self-reported conditions associated with MASLD, including liver disease (ie, hepatitis, hemochromatosis), chronic kidney disease, diabetes, regular or excessive use of alcohol (defined as >30 grams per day for males and >15 grams per day for females), and current use of medications that cause steatosis (ie, corticosteroids). We received institutional review board approval from The Queen's Medical Center Research & Institutional Review Committee in Honolulu, Hawai’i.

### Study Procedures

Each participant attended a single scheduled visit at a designated community clinic and completed 2 questionnaires: 1 to screen inclusion/exclusion criteria and the Beverage Intake Questionnaire, a valid and reliable self-reported beverage intake questionnaire that quantifies the consumption of sugar-sweetened beverages (SSB) [19].

Participants' height, weight, waist and hip circumference, and systolic and diastolic blood pressure were measured. Waist circumference was measured at the horizontal plane midway between the lowest rib and the iliac crest. Hip circumference was taken at the widest circumference around the greater trochanters. Systolic and diastolic blood pressure were obtained in duplicate and averaged using an automated sphygmomanometer (Omron HEM-907XL) after the participant remained seated for 5 minutes [20].

Blood specimens were collected by trained technicians and processed at a local commercial laboratory (Diagnostic Laboratory Services, Inc.) after a minimal 8-hour fast to measure serum cholesterol, triglycerides, high density lipoprotein, calculated low density lipoprotein, C-peptide, hemoglobin A1c, fasting glucose, alanine aminotransferase, and aspartate aminotransferase. All laboratory analyses were performed according to equipment manufacturers' recommendations.

Each participant underwent liver ultrasonographic vibration-controlled transient elastography (VCTE) with controlled attenuation parameter (CAP) using the FibroScan 430 (Echosens, Paris, France), a noninvasive Food and Drug Administration-approved method to measure steatosis and fibrosis in patients with MASLD [21]. VCTE is a reproducible method to assess liver steatosis with a sensitivity and specificity of 89% [22] and 93% [23], respectively. A CAP cutoff score ≥238 dB/m was used to identify MASLD based on an evaluation of multiple studies [22, 24-27].

### Statistical Analysis

An initial power analysis could not be performed due to the absence of preliminary prevalence data in Hawai’i's MASLD population. In this pilot study, a convenience sample of 100 individuals were evaluated. Descriptive statistics were presented in the forms of mean and standard deviations for continuous data and frequencies for categorical data. We performed chi-square tests to compare the distribution of categorical variables between the non-MASLD (CAP < 238 dB/m) and MASLD groups (CAP ≥ 238 dB/m) and to determine if a relationship exists between MASLD and obesity status. Kolmogorov-Smirnov test was used to evaluate for normal distribution of continuous variables. To compare the distribution of continuous variables between the non-MASLD and MASLD groups, the Mann-Whitney *U*-test was used for nonparametric data and for parametric continuous variables, Student's *t*-tests were used. The *P*-values for all statistical analysis were 2-tailed, with a significance level of .05. To investigate associations between age, BMI, CAP score, and SSB consumption, we used Spearman's Rho. We performed all analyses using IBM SPSS Statistics for Windows, Version 28.

## Results

### Clinical Characteristics of the Study Population

One hundred participants were enrolled with a mean age of age 25.4 (5.5) years with 68% female, 46% NHOPI, and 36% Asian. An overall 44% of participants had MASLD, defined by a CAP ≥ 238. No significant difference was noted for age, sex, or race between the non-MASLD and MASLD groups. Those with MASLD had a significantly higher fibrosis score, based on VTCE, than those without MASLD, though remaining within normal limits (6.0 vs 5.1 kPa, respectively, *P* = .022), with a normal score being <7.9 kPa [28]. Those with MASLD had a higher BMI compared to those without MASLD, 30.2 (5.5) vs 23.2 (3.6), respectively (*P* < .001) as well as higher systolic blood pressure of 126.6 (9.7) mmHg vs 118.3 (11.9) mmHg, (*P* < .001), higher waist-to-hip ratio of 0.90 (0.06) vs 0.82 (0.07), (*P* < .001), and higher waist-to-height ratio of 0.58 (0.08) vs 0.46 (0.06), (*P* < .001) (Table 1). Mean alanine aminotransferase levels in the non-MASLD group were 15.8 Iu/L while those with MASLD were significantly higher at 35.5 Iu/L (*P* < .001). A total of 19 continuous variables were statistically significant (defined as *P* < .05) between those with and without MASLD. Incidentally, 8 participants were diagnosed with diabetes based on a hemoglobin A1c (HbA1c) result ≥6.5%.

**Table 1.** Population comparison between non-MASLD and MASLD in adolescent and young adults

	All Participants n = 100	Non-MASLD n = 56	MASLD n = 44	*P*-value
Sex				.29
Female	68	41 (73.2%)	27 (61.4%)	
Ethnicity				.97
Asian	36	21 (37.5%)	15 (34.1%)	
NHOPI Filipino	46	25 (44.6%)	21 (47.7%)	
Other	18	10 (17.9%)	8 (18.2%)	
	All Participants	Non-MASLD Mean (SD)	MASLD Mean (SD)	*P*-value
Age, years	25.4 (5.5)	24.9 (5.5)	26.1 (5.5)	.29
BMI	26.3 (5.7)	23.2 (3.6)	30.2 (5.5)	<.001
SBP	121.9 (11.7)	118.3 (11.9)	126.6 (9.7)	<.001
DBP	78.6 (9.1)	74.8 (7.7)	83.4 (8.5)	<.001
Waist Circumference, in.	33.2 (6.0)	29.5 (3.5)	37.8 (5.2)	<.001
HipCircumference, in.	38.8 (5.2)	36.1 (3.8)	42.2 (4.7)	<.001
Waist-hip ratio	0.85 (0.07)	0.82 (0.07)	0.90 (0.06)	<.001
Waist-height ratio	0.51 (0.09)	0.46 (0.06)	0.58 (0.08)	<.001
CAP, dB_M	227.1 (62.6)	182 (31.4)	284.5 (41.9)	<.001
Fibrosis, kPa	5.5 (2.2)	5.1 (1.5)	6.0 (2.8)	.062
Cholesterol, mg/dL	180.1 (31.5)	176.1 (29.5)	185.1 (33.5)	.17
Triglycerides, mg/dL	111.1 (162.5)	78.6 (43.1)	152.4 (235.2)	<.05
HDL, mg/dL	57.9 (14.8)	64.8 (13.7)	49.1 (11.1)	<.001
LDLc, mg/dL	102.9 (26.7)	95.5 (24.2)	112.4 (27.0)	<.001
nonHDLc, mg/dL	122.2 (33.1)	111.3 (27.6)	136.1 (34.5)	<.001
C-Peptide, ng/mL	2.3 (1.2)	1.9 (1.1)	2.9 (1.1)	<.001
cPep Index	6.7 (6.3)	7.8 (4.0)	5.4 (8.1)	.072
cPep Index_b	2.4 (1.2)	2.1 (1.0)	2.9 (1.3)	<.001
HbA1c, %	5.7 (1.2)	5.5 (0.9)	5.9 (1.6)	.13
Fasting glucose, mg/dL	101.4 (40.6)	95.8 (34.7)	108.5 (46.5)	.14
Trigly-Glu Index	4.5 (0.3)	4.4 (0.2)	4.7 (0.4)	<.001
AST, Iu/L	20.6 (8.9)	18.9 (7)	22.8 (10.5)	.004
ALT, Iu/L	24.5 (20.6)	15.8 (7.1)	35.5 (26.2)	<.001
AlkalinePhos, Iu_L	77.4 (37.7)	78.7 (45.8)	75.8 (24.2)	.64
TotalProtein, mg_dL	7.2 (0.4)	7.2 (0.4)	7.3 (0.4)	.48
Albumin, mg_dL	4.7 (0.3)	4.7 (0.3)	4.7 (0.2)	.47
DailySSB, fl. Oz	10.8 (25.7)	6.3 (9.5)	16.5 (36.5)	.079
DailySSB, kCal	134.1 (339.1)	75.2 (123.8)	209.1 (484.7)	.081

Abbreviations: ALT, alanine aminotransferase; AST, aspartate aminotransferase; BMI, body mass index; CAP, controlled attenuation parameter score; DBP, diastolic blood pressure; HDL, high-density lipoprotein; HbA1c, glycated hemoglobin A1c; LDLc, calculated low-density protein; MASLD, metabolic-associated steatotic liver disease; NHOPI, Native Hawaiian and other Pacific Islanders; SBP, systolic blood pressure; SSB, sugar-sweetened beverage measured by fluid oz (fl_oz) intake and calorie intake (kCal); TC, total cholesterol; TG, triglyceride.

Age was measured in years; ethnicity was self-reported as “most identified as.”

Waist and circumference was measured in inches; waist-hip ratio was calculated by dividing waist measurements to hip; waist-height ratio was calculated by dividing waist measurements to height.

### Prevalence of MASLD vs non-MASLD by Obesity Status

Of the 100 participants, 74 were nonobese of which 31.1% [95% confidence interval (CI): 20.5-41.6%] had MASLD and 68.9% did not have MASLD (Table 2). Of the 26 obese participants, 80.8% (95% CI: 65.6-95.9%) had MASLD and 19.2% did not have MASLD. The chi-squared test results were statistically significant.

**Table 2.** Chi-squared test comparing MASLD and non-MASLD in individuals with BMI < 30 and BMI ≥ 30

	Nonobese (n = 74) n (%)	Obese (n = 26) n (%)	*P*-value
MASLD	23 (31.0)	21 (80.8)	<.001
Non-MASLD	51 (68.9)	5 (19.2)	

Abbreviations: BMI, body mass index; MASLD, metabolic-associated steatotic liver disease.

Nonobese (BMI < 30); obese (BMI ≥ 30).

### Nonobese vs Obese in Those With MASLD

Of the 44 participants with MASLD, 52.3% (95% CI: 36.7-67.5%) were nonobese and 47.7% (95% CI: 32.5-63.3%) were obese (Table 3). Of the nonobese MASLD, 43.4% were Asian, 34.8% NHOPI, and 21.7% White and other. Of participants with obese MASLD, 23.8% were Asian, 61.9% NHOPI, and 14.3% White and other. In those with MASLD, based on obesity status and ethnicity, the results were not statistically significant (*P* = .19).

**Table 3.** MASLD group by obesity status according to ethnicity

	Nonobese MASLD n (%)	Obese MASLD n (%)	*P*-value
All	23 (52.3)	21 (47.7)	
Ethnicity			
Asian*^a^*	10 (43.4)	5 (23.8)	
NHOPI	8 (34.8)	13 (61.9)	.19
White and other	5 (21.7)	3 (14.3)	

Abbreviations: MASLD, metabolic-associated steatotic liver disease; NHOPI, Native Hawaiian and other Pacific Islanders.

Nonobese MASLD (BMI < 30); obese MASLD (BMI ≥ 30).

^*a*^When the Asian obesity cutoff is applied, the distribution of nonobese (BMI < 25) MASLD is n = 4 (23.5%) and obese (BMI ≥ 25) MASLD n = 11 (40.7%). The *P*-value changes to .243 from .19.

### MASLD Associated WithSSB Intake

**Figure 1.**
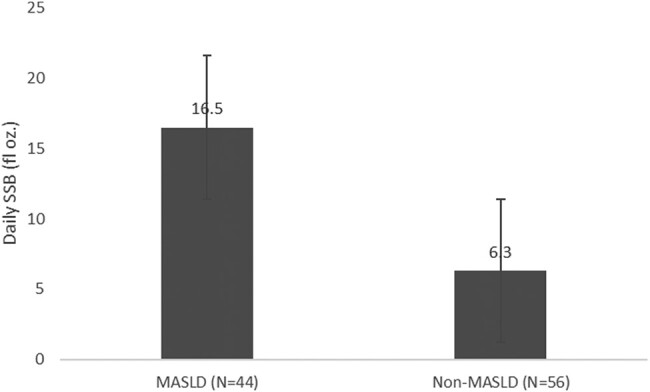
Mean daily SSB volume differences between MASLD and non-MASLD.

Abbreviations: MASLD, metabolic-associated steatotic liver disease; SSB, sugar-sweet beverage.

**Figure 2.**
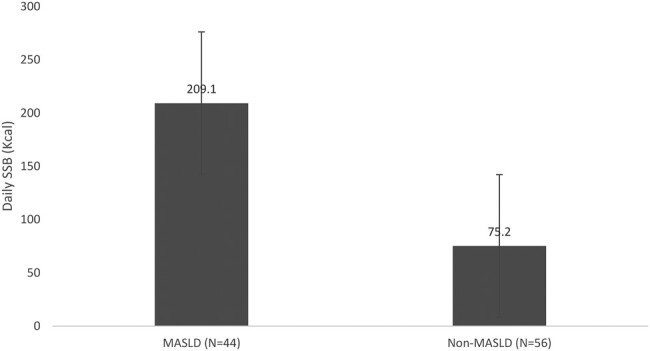
SSB kCal differences between MASLD and non-MASLD.

Abbreviations: MASLD, metabolic-associated steatotic liver disease; SSB, sugar-sweet beverage.

The results from the Beverage Intake Questionnaire survey showed that AYA without MASLD drank on average 6.3 fluid oz (95% CI: 3.7-8.8) and 75.2 kCal (95% CI: 42.1-108.4) (*P* = .079) of SSB compared to 16.5 fluid oz (95% CI: 5.3-27.6) and 209.1 kCal (95% CI: 61.7-356.5) (*P* = .081) in those with MASLD (Figs. 1 and 2). While there was a strong trend, results were not significant (Table 1).

### Correlation Studies With age, BMI, CAP, and Daily SSB (Kcal) Consumption

Correlation studies showed that there were statistically significant associations between BMI and CAP (r = .750, *P* = <.001), BMI and daily SSB (kcal) (r = .206, *P* = .040), and CAP and daily SSB (kcal) consumption (r = .235, *P* = .018) (Table 4). Although not statistically significant, there was a negative correlation with age and daily SSB (kcal) (r = −0.068 *P* = .500).

**Table 4.** Correlation studies with age, BMI, CAP, and daily SSB consumption

	Age	BMI	CAP score	Daily SSB (kCal)
Age	1			
BMI	0.184	1		
	*P* = .067			
CAP score	0.158	0.750*	1	
	*P* *=* .117	*P* < .001		
Daily SSB (kCal)	−0.068	0.206*	0.235*	1
	*P* = .5	*P* *=* .04	*P* *=* .018	

Abbreviations: BMI, body mass index; CAP, controlled attenuation parameter; SSB, sugar-sweetened beverages.

^*^Correlation is significant at the .05 level (2-tailed).

## Discussion

MASLD is known to have a high prevalence in those with T2DM and obesity. Here, this cross-sectional pilot study evaluated a young cohort of Hawai’i residents without a known history of diabetes who would have typically been considered healthy and free of liver disease including MASLD. Secondary causes of liver disease (ie, hemochromatosis, hepatitis, Wilson disease, etc.) were not evaluated due to funding restraints and should be considered in future studies. As part of the FibroScan^®^, a fibrosis score is reported. A significantly higher, yet within normal limits, score was found in those with MASLD compared to those without MASLD. Potentially, the addition of the fibrosis—4 score may assist in identifying hepatic fibrosis but may miss early disease. Instead, we relied on liver VCTE with a CAP score of ≥238 dB/m to identify MASLD; however, there is no consensus on a clear CAP cutoff to identify MASLD [21]. A more in-depth evaluation will help determine the sensitivity and specificity of the CAP used and which cutoffs are better suitable in this relatively unstudied population in Hawai’i.

These results establish a foundation for larger studies to deepen our understanding of MASLD in Hawai’i. Our sample size of 100 seemingly healthy participants (82% identifying as NHOPI or Asian) identified a prevalence of 44%, which is markedly greater than the reported children, adolescent, adult U.S., and worldwide prevalence of MASLD. At this time, the natural history of MASLD in Hawai’i is unknown, and further studies are needed to help stratify between the MASLD subgroups, SLD and MASH, and the age and incidence of those eventually requiring liver transplantation.

**Figure 3.**
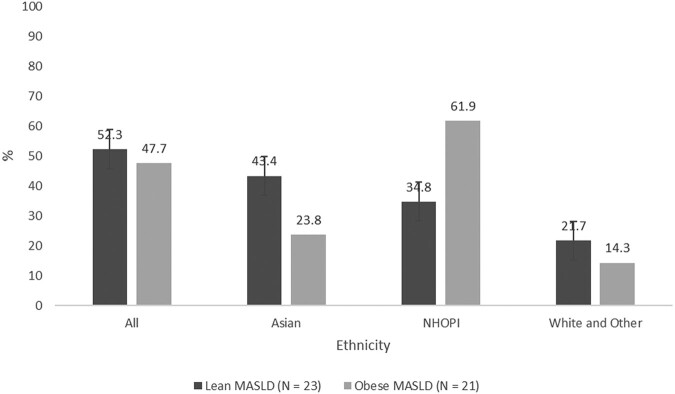
MASLD group by obesity status according to ethnicity.

Abbreviations: MASLD, metabolic-associated steatotic liver disease.

In the Hawai`i sample, there was a higher MASLD prevalence in obese compared to nonobese participants (*P* < .001) (Table 2). To gain further understanding and establish trends, we evaluated the 44 individuals with MASLD. In those with MASLD, although not statistically significant, there was a slightly higher overall MASLD rate in the nonobese group (52.3%) compared to the obese cohort (47.7%) (Fig. 3). When stratifying by ethnicity, Asians dominated the nonobese MASLD group (43.4%), while NHOPIs led the obese MASLD group (61.9%). Recent studies suggest that nonobese MASLD individuals have more severe hepatic disease progression and higher mortality compared to their obese MASLD counterparts [7, 29-33]. These findings are concerning especially in regions with robust Asian populations such as Hawai`i. The burden of nonobese MASLD highlights the need for guidelines that account for other factors and do not exclusively include BMI as a criterion for MASLD screening.

A potential consideration for the higher proportion of MASLD in nonobese Asians may be related to the use of standard BMI of ≥30 to identify obesity for all ethnic groups, while a cutoff point of ≥25 has been suggested to be more representative of obesity in Asians [34]. Reanalyzing data from the Asian subgroup and adjusting for a BMI ≥ 25.0, the proportion of nonobese Asians with MASLD fell to 23.5% (BMI < 25.0) from 43.4% (BMI < 30) and the obese MASLD group increased to 40.7% (BMI ≥ 25.0) from 23.8% (BMI ≥ 30) (Table 3). Even with this adjustment, the association between obese and nonobese remained nonsignificant (*P*-value from .19 to .243) with ethnicity. At this time, it is unclear which BMI cutoff values are ideal for Asians who grew up in Hawai’i considering that, while identifying as “Asian,” many are of mixed ethnic backgrounds. As an alternative to BMI, studies suggest the use of waist-to-hip ratio or waist-to-height ratio is a better determinant of cardiometabolic risks in different ethnic populations [35-39]. In our study, those with MASLD showed higher waist-to-hip ratio compared to non-MASLD (0.90 vs 0.82, respectively, *P* < .001) as well as waist-to-height ratios (0.58 vs 0.46, respectively, *P* < .001).

Moreover, within the Hawai’i Asian cohort, 41.7% had MASLD, similar to the general Asian American prevalence of 43.2% [40] but higher than the 29.6% MASLD overall prevalence of Asians living in Asia [41]. This could be related to significant dietary differences between Western diet and traditional foods in Asia and the increased consumption of SSB, a known risk factor for the development of MASLD [42, 43]. This study shows a strong trend suggesting that those with MASLD consume nearly 3 times more SSB compared to those without MASLD, which is also consistent with the literature [44]. Larger studies are required to validate this data, and, if significance is identified, then more education on health outcomes associated with SSB consumption on both population and clinical levels may be indicated.

Correlation studies resulted as expected showing associations between BMI and CAP, BMI and daily SSB consumption, and CAP and daily SSB consumption (Table 4). While not statistically significant, a negative association between age and SSB consumption was noted, which is consistent with studies showing that teenagers consume the highest amounts of SSB relative to other age groups [45].

Incidentally, biometric results showed 8% of participants had a HbA1c ≥ 6.5% and were diagnosed with diabetes as a result of this study. All 8 participants were from community recruitment; 3 had an HbA1c level ≥ 11%. These findings suggest that there is a considerable undiagnosed population with diabetes living in Hawai’i.

As a globally increasing problem and a leading cause of liver disease and HCC in adults receiving liver transplants [46], along with a relatively high rate of uninsured NHOPI in Hawai’i [47], further stratification of the NHOPI population is indicated for early identification of MASLD to promote early intervention. In those with MASLD, the NHOPI obese group is of particular interest with its 61.9% prevalence of MASLD. The rapid Westernization of diet is likely a leading cause of the obesity epidemic in the NHOPI and Asian populations in Hawai’i. A return to a traditional diet has been shown to improve cardiometabolic risks in Native Hawaiians [48], though this is an unlikely sole solution and other modalities need exploration.

## Conclusions

Our pilot study shows an overall 44% prevalence of MASLD in a young cohort of Native Hawaiians, other Pacific Islanders, and Asians living in Hawai’i that appears to be disproportionately higher than those in other parts of the United States and may be correlated, in part, to high consumption of SSB.

## Summary

To date, this is the first study in Hawai’i that has evaluated a healthy cohort of AYA to assess the prevalence of MASLD with VCTE and showed an overall 44% prevalence of MASLD. Of those who were identified with MASLD, none were aware, which is consistent with the low screening rates seen globally [14] and in Hawai’i.

Studies that incorporate a large randomized sample will help to broaden our epidemiological understanding of MASLD in Hawai’i, while more directed studies can stratify between the different subtypes (SLD and MASH) and help predict future increased health care needs and costs associated with HCC and liver transplants. In the long term, this will bring more awareness of a condition that is commonly missed though has significant repercussions including liver failure, cancer, and need for liver transplantation.

## Data Availability

Some or all datasets generated during and/or analyzed during the current study are not publicly available but are available from the corresponding author on reasonable request.
